# Long-Term Growth Hormone Treatment of Children with PWS: The Earlier the Start, the Better the Outcomes?

**DOI:** 10.3390/jcm11092496

**Published:** 2022-04-29

**Authors:** Lionne N. Grootjen, Demi J. Trueba-Timmermans, Layla Damen, Eva F. Mahabier, Gerthe F. Kerkhof, Anita C. S. Hokken-Koelega

**Affiliations:** 1 Dutch Reference Center for Prader-Willi Syndrome, 3015 CN Rotterdam, The Netherlands; d.timmermans@kindengroei.nl (D.J.T.-T.); l.damen@kindengroei.nl (L.D.); e.mahabier@kindengroei.nl (E.F.M.); g.kerkhof@erasmusmc.nl (G.F.K.); a.hokken@erasmusmc.nl (A.C.S.H.-K.); 2 Department of Pediatrics, Subdivision of Endocrinology, Erasmus University Medical Center-Sophia Children’s Hospital, 3015 CN Rotterdam, The Netherlands; 3 Dutch Growth Research Foundation, 3016 AH Rotterdam, The Netherlands

**Keywords:** Prader-Willi syndrome, children, body composition, cognition, growth hormone

## Abstract

Long-term effects of growth hormone (GH) treatment in young children with Prader-Willi syndrome (PWS) have never been compared with untreated age-matched controls with PWS, and it is unclear if starting GH in the first year of life is safe and more effective than starting GH in early childhood. We investigated the effects of long-term GH on body composition, anthropometrics and cognition in young children with PWS compared to untreated controls and assessed whether starting GH in the first year of life is optimal and safe. An open-label, prospective study was performed, comparing GH-treated children with untreated controls, and comparing children who started GH in the first year of life (subgroup A) with children who started between 2–5 years (subgroup C). A total of 82 GH-treated children with PWS and 22 age-matched controls with PWS were included. The main outcome measures were body composition, anthropometrics, IQ, and safety parameters. After 8 years, GH-treated children had significantly better body composition and were taller than age-matched controls. Subgroup A had a lower FM% trajectory during treatment than subgroup C and showed a greater and longer-term increase in the LBM index. After 8 years, subgroup A had a lower trunk/peripheral fat ratio (*p* = 0.043) and higher IQ (*p* = 0.043). No adverse effects of starting GH in the first year were found. Children with PWS who received long-term GH had a better body composition and growth than untreated age-matched controls and starting GH in the first year of life was optimal and safe.

## 1. Introduction

Prader-Willi syndrome (PWS) is a rare syndrome caused by the lack of expression of genes in the PWS region on the paternally derived chromosome 15, caused by a paternal deletion, maternal uniparental disomy (mUPD), or an imprinting-centre defect (ICD) or paternal chromosomal translocation [1,2]. Clinical findings characterising PWS are abnormal body composition, muscular hypotonia, developmental delay, behavioural problems, hyperphagia, obesity, and short stature [2].

Studies have shown that GH treatment of children with PWS improves body composition, linear growth and cognition [3,4,5,6,7]. GH treatment was approved for children with PWS in 2001, but the effects of long-term GH treatment of children with PWS have never been compared with age-matched untreated controls.

Because of improved genetic testing and early recognition of the clinical characteristics, PWS is nowadays often diagnosed in the first few weeks of life. This has provided the opportunity to start multidisciplinary care and GH treatment in early life. A retrospective and a post-marketing study found that starting GH treatment in early life has positive effects on height SDS and BMI SDS [8,9]. However, to our knowledge, there is no prospective study investigating if it is safe and more beneficial to start GH treatment in the first year of life compared to starting between age 2–5 years, regarding body composition, anthropometric parameters, and cognitive functioning.

The primary objective of our study was to investigate the effects of long-term GH treatment on body composition, anthropometric parameters, and cognitive functioning in children with PWS compared to age-matched untreated controls with PWS. The secondary objective was to investigate whether children who start GH treatment in the first year of life have significantly better body composition, anthropometric parameters, and cognition after 8 years of GH treatment, compared to those starting between the age of 2–5 years. Lastly, we aimed to determine long-term safety of GH treatment when started in the first year of life. We hypothesised that children treated with GH for 8 years would have a significantly better body composition, anthropometric parameters, and cognition compared to age-matched untreated controls. We also hypothesised that starting GH treatment in the first year of life is safe and results in significantly better body composition, height, and cognition after 8 years of GH treatment than starting between the age of 2–5 years.

## 2. Methods

### 2.1. Patients and Controls

All participants were diagnosed with PWS, confirmed by methylation analysis of the PWS region, and participated in the Dutch PWS Cohort study [3,6]. All children were studied from start of their GH treatment. Exclusion criteria of present study were: less than 8 years of continuous GH treatment, presence of obstructive sleep apnoea diagnosed with polysomnography, and age above 5.0 years at start of GH treatment. Twenty-seven were also included in a previous study [6]. The untreated control group consisted of age-matched children with PWS, who participated in our randomised controlled trial [3]. The years of birth of the GH-treated children range from 1998 to 2012 and the date of birth of the control group ranged from 1990–1998.

Pubertal stage was defined as prepubertal (testes volume < 4 mL or Tanner breast stage < 2), early pubertal (testes volume 4–8 mL or Tanner breast stage 2), mid pubertal (testes volume 8–12 or Tanner breast stage 3), or late pubertal (testes volume > 12 mL or Tanner breast stage ≥ 4) [10].

### 2.2. Design

In this prospective study, all children in the GH group were treated with 1.0 mg GH/m^2^/day (~0.035 mg/kg) for 8 consecutive years. At each visit, GH dose was adjusted to body surface area. Before initiation of GH treatment, all children underwent polysomnography (PSG).

All children visited the Dutch PWS Reference Center in Rotterdam and received multidisciplinary care from the PWS team in collaboration with paediatric endocrinologists and paediatricians in other Dutch hospitals. The Dutch PWS Cohort study (MEC-2001-230, 18 September 2001) was approved by Medical Ethics Committee of the Erasmus University Medical Center. Written informed consent was obtained from parents and children older than 12 years. Assent was obtained from children younger than 12 years. The study was conducted according to the guidelines of the Declaration of Helsinki II.

### 2.3. Dual Energy X-Ray Absorptiometry (DXA)

DXA-scans (Lunar Prodigy type; GE healthcare, Chalfont St. Giles, UK) were annually performed to measure lean body mass (LBM) and fat mass percentage (FM%). The DXA-machine was calibrated daily, and all scans were performed with the same machine. The intra-assay coefficients of variation were 0.41–0.88% for fat tissue and 1.57–4.49% for LBM [11]. LBM index (LBMI) was calculated as LBM (kg)/squared height (meters).

### 2.4. Anthropometrics

Standing height was measured with Harpenden Stadiometer and supine length with Harpenden Infantometer (Holtain Ltd., Crosswell, UK). Measurements were performed by the PWS team. Weight was measured on a calibrated scale (Servo Balance KA-20-150S; Servo Berkel Prior, Katwijk, The Netherlands). Height, weight, and body mass index (BMI) standard deviation scores were calculated with Growth Analyser RCT 4.1, based on Dutch Reference values [12,13].

### 2.5. Cognition

Wechsler Intelligence Scale for Children (WISC) was used to assess cognitive functioning. The WISC is suitable for children between the age of 6 and 16 years [14]. Because of limited attention span of children with PWS, the subtests Block Design, Vocabulary, and Similarities were used. Results of this short version of the WISC are correlated with the full-scale IQ test [15,16,17]. Calculation of total IQ (TIQ) was performed using: TIQ = 45.3 + 2.91 × vocabulary Sub Score (SS) + 2.5 × Block design SS. This formula has been used in other studies and is based on a Dutch reference population [18,19,20].

### 2.6. Assay

Fasting blood samples were collected for assessment of fasting serum levels of IGF-1, insulin, glucose, triglycerides, total cholesterol, HDL, and LDL. Blood samples were measured in the Biochemical and Endocrine laboratories of the Erasmus University Medical Center, Rotterdam. The different assays with their intra- and inter-assay CVs are described elsewhere [21]. Because serum IGF-1 levels are age- and sex-dependent, values were transformed to SDS values, based on Dutch population [22]. Homeostatic model assessment of insulin resistance (HOMA-IR) was performed using the model HOMA-IR = (fasting insulin × fasting glucose)/22.5 [23].

### 2.7. Blood Pressure

Systolic and diastolic blood pressure (BP) were measured using an appropriately sized cuff while patients were in sitting position. Because height and sex are important determinants of BP in childhood, BP was expressed as SDS adjusted for height and gender [24]. None of the children were receiving antihypertensive therapy.

### 2.8. Bone Maturation

Radiographs of the left hand were taken. Bone age (BA) was assessed in duplo by two independent observers (L.N.G. and D.J.T.) based on Greulich and Pyle [25]. The mean (SD) difference between observers was 0.33 (0.35) years. Mean BA was used for further analysis. The bone-age-to-calendar-age ratio (BA/CA) was calculated and used in analysis.

### 2.9. Statistics

Statistical analyses were performed with SPSS 24.0 (SPSS INC, Chicago, IL, USA). Distribution of variables was determined by Kolmogorov–Smirnov test and Q–Q plots. Clinical characteristics are presented as mean (SD). GH-treated children were divided into three subgroups based on tertiles of age at start of GH: subgroup A (age at GH start between 0–1.05 years), subgroup B (age at GH start between 1.05–2.27 years), and subgroup C (age at GH start between 2.27–5 years). We compared the lowest tertile with the highest tertile to achieve the highest statistic power. Differences between GH-treated children and untreated controls and between subgroups A and C were evaluated using an independent-sample *t*-test or chi-square test. FM% SDS and LBM SDS after 8 years of GH treatment were calculated according to age- and sex-matched Dutch reference values [26]. Since height SDS was very different between GH-treated children and untreated controls, FM% SDS, FM%, trunk fat vs. peripheral fat ratio and LBM SDS were additionally corrected for height and analysed with ANCOVA for the comparison of the GH-treated group with untreated controls. As pubertal stage differed between subgroup A and C, FM%, trunk vs. peripheral fat ratio, and the metabolic parameters were corrected for sex and pubertal stage and ANCOVA was used for analyses. All ANCOVA results are presented as Estimated Marginal Mean (EMM) (standard error of the mean (SEM)). Since there are no reference values to calculate SDS for FM% and LBM before the age of 4 years, we determined the trajectories of FM% and LBMI, both corrected for sex and pubertal stage. Differences in these trajectories over time between the two subgroups were tested with linear mixed models with years of GH treatment as repeated variable and an unstructured covariance matrix. Results of the linear mixed models are presented as EMM (SEM). Level of significance was set at a *p*-value of 0.05.

## 3. Results

One hundred and fifty-two children started GH treatment before 1 June 2013. Fifty-six of them were older than 5 years of age at the start of GH treatment and 14 were lost to follow-up. In total, 82 children started GH before the age of 5 years and completed at least 8 years of continuous GH treatment. The control group consisted of 22 age-matched untreated children with PWS.

### 3.1. Baseline Characteristics

Table 1 shows the baseline characteristics at start of GH treatment. No difference in genetic subtype was found between GH-treated group and untreated controls. Target height SDS was not different between the GH-treated children and controls. Subgroup A consisted of 27 children with a mean (SD) age of 0.75 (0.13) years at GH start, and subgroup C of 27 children, with a mean (SD) age of 3.38 (1.2) years at GH start. There was no difference in genotype between subgroups. Infants in subgroup A had significantly lower weight for height SDS (*p* = 0.017) and tended to be taller (*p* = 0.064) at start of GH treatment. There was no difference in body composition, except for a lower trunk/peripheral fat ratio in the infants of subgroup A, who were below the age of 1 year (*p* = 0.001). Target height SDS was not different between subgroup A and C.

### 3.2. Results after 8 Years of GH Treatment Compared to Age-Matched Untreated Controls

Table 2 presents the results of the GH-treated group compared to untreated controls. The mean (SD) age of GH-treated children and untreated controls was similar. No significant difference in pubertal stage was found between the groups. The mean FM% was 38.0% in the GH-treated group and 46.1% in the untreated controls (*p* < 0.001). The mean FM% SDS was also significantly lower in the GH-treated group (*p* = 0.036) and trunk/peripheral fat ratio tended to be lower (*p* = 0.065). Children treated with GH had a higher LBM SDS (*p* = 0.033 (corrected for height)) and were significantly taller (*p* < 0.001) compared to untreated controls. The GH-treated children had a larger head circumference (*p* < 0.001) compared to untreated controls, and the GH-treated children scored higher on every subtest and had a higher total IQ, but this did not reach significance.

### 3.3. Results of 8 Years of GH Treatment in Subgroup A versus C

Table 3 presents the results of the three subgroups after 8 years of GH treatment. After 8 years, subgroup A (GH started in the first year of life) had a mean (SD) age of 8.75 (0.19) years and subgroup C (GH started between age 2–5 years) had a mean (SD) age of 11.33 (0.80) years (*p* < 0.001). None of the children had a late pubertal stage.

### 3.4. Body Composition

**Figure 1:**
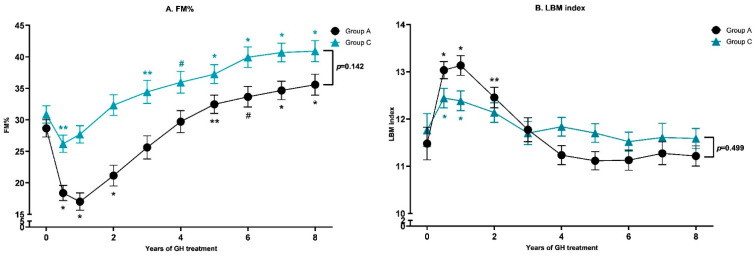
Longitudinal changes in Estimated Marginal Means with SEM in FM% (**A**) and LBM index (**B**) in group A (27 children with PWS, age start GH treatment < 1.05 years) and group C (27 children with PWS, age start GH treatment between 2.27–5.00 years) during 8 years of GH treatment. FM% and LBMI are corrected for sex and pubertal stage. * *p*-value < 0.001, ^#^ *p*-value < 0.01, ** *p*-value < 0.05. All these *p*-values are compared to baseline, *p*-values indicated by the brackets are referring to the difference between the subgroups after 8 years.

After 8 years of GH, FM% SDS was 1.81 (0.54) in subgroup A and 2.06 (0.87) in subgroup C (*p* = 0.212). In addition, mean (SD) trunk/peripheral fat ratio was, after 8 years of treatment, lower in subgroup A than in subgroup C (*p* = 0.043) (Table 3). The trajectory of FM% (corrected for sex and pubertal stage) during 8 years of GH was lower in children who started GH in the first year of life (*p* < 0.001) (Figure 1A). In these children, FM% decreased considerably after the start of GH treatment (*p* < 0.001) and this decrease was less strong in children who started GH between 2–5 years. After 8 years of GH, FM% was 36.0 (1.70) % in subgroup A and 40.7 (1.78) % in subgroup C (*p* = 0.142).

Figure 1B shows the trajectory of LBMI during 8 years of GH treatment. The difference between the trajectory of subgroup A and C was significant (*p* < 0.001). In both subgroups, an increase in LBMI was found after the start of GH, but the increase in subgroup A persisted for a longer time, leading to higher LBMI during the first few years of treatment. After 8 years of GH treatment, the LBMI between the subgroups was comparable.

### 3.5. Anthropometry

After 8 years of GH, height SDS was not significantly different between the subgroups (*p* = 0.199) and within the normal range for both subgroups. Head circumference SDS did not differ between the subgroups (Table 3).

### 3.6. Cognition

After 8 years of GH treatment, children in subgroup A scored significantly higher in the Vocabulary subtest, with a mean (SD) SS (subscore) of 6.71 (2.56) in subgroup A, and 3.63 (2.54) in subgroup C (*p* = 0.012) (Table 3). The SS in Block Design and Similarities were not different between the subgroups, but the mean (SD) Total IQ was significantly different: 78.1 (12.6) in subgroup A and 64.8 (14.5) in subgroup C (*p* = 0.043).

### 3.7. Safety Parameters

Table 4 shows the safety parameters after 8 years of GH treatment. No differences in fasting serum levels of glucose, insulin, and triglyceride were found between subgroups (all *p*-values > 0.207). Cholesterol was lower in children who started GH in the first year of life compared to those started between 2–5 years (*p* = 041). Mean (SD) systolic BP SDS was higher in subgroup A (0.72 (0.89)) than in subgroup C (−0.06 (0.88)), but in all children, systolic BP SDS was within the normal range. Diastolic BP did not differ between subgroups.

## 4. Discussion

This is the first study investigating the long-term effects of GH on body composition, anthropometrics, cognition, and safety parameters in a large cohort of children with PWS in comparison with age-matched untreated controls with PWS. In addition, we compared the effects of 8 years of GH between two subgroups, based on age when starting GH. Our findings demonstrate that 8 years of GH resulted in better body composition, taller stature, and larger head circumference in children with PWS compared to age-matched untreated controls. In addition, we found that children who started GH before the age of 1 year had a significantly lower FM% trajectory over an 8-year period than children who started between 2–5 years, with a more prominent decrease in FM% during the first few years of treatment, and also a marked increase in LBMI in these first few years. In addition, these children had a more favourable trunk/peripheral fat ratio and a tendency to a lower FM% after 8 years of GH compared to children who were 2–5 years at the start of GH, without differences in safety parameters. Starting GH in the first year of life was associated with a higher total IQ after 8 years of GH treatment. Our findings might suggest that it may be most optimal to start with GH in the first year of life.

After 8 years of GH treatment, children with PWS had lower FM% SDS and higher LBM SDS than age-matched untreated controls with PWS, which is in line with our hypothesis. Thus, our findings show that 8 years of GH in children with PWS who started GH before the age of 5 years resulted in better body composition compared to age-matched untreated children with PWS.

In addition, we found a lower fat mass % trajectory in the children who started GH in the first year of life compared to those who started between age 2–5 years. This was mainly due to a more prominent decrease in FM% after starting GH in the first year of treatment in children who started GH in the first year of life, while the decrease was less strong in those who started GH between age 2–5 years. FM% after 8 years of GH was also lower in the group that started treatment before the age of 1 year, although this did not reach significance, probably due to relatively low numbers in the subgroups. As the number of pubertal children in the subgroup that started GH in the first year of life was smaller than in the other subgroup, we adjusted for pubertal stage. One retrospective study investigated the effect of age at start of GH on body composition during 5 years of treatment, but found no relation with body fat [8]. This difference may be due to their use of bioimpedance spectroscopy for body composition analysis, instead of DXA-scan. Another explanation could be the much lower IGF-1 SDS in their study, likely caused by the lower GH dose of 0.0289–0.0263 mg/kg/day, while our study population used the recommended GH dose for PWS: 1 mg/m^2^/day (~0.035 mg/kg/day) [2]. It has been shown that a lower GH dose does not reduce FM in PWS [27]. Our findings show that starting GH in the first year results in a strong decrease in FM% during the first year of life. As accelerated gain in FM in the first six months of life is, in general, associated with more adiposity at the age of 2 years [28], it might be important to start GH as early as possible, preferably during this critical window for adiposity programming in the first year of life.

Children who started GH in the first year of life had also a more healthy trunk/peripheral fat ratio than those who started between age 2–5 years. This is in line with the trend found towards a lower FM% in the children who started GH in the first year of life. It might be that starting GH in the first year of life prevents the deterioration in fat distribution. A lower trunk/peripheral fat ratio after 8 years is clinically relevant, as central obesity is a predictor of cardiometabolic disease, even more so than general obesity [29], and it is associated with insulin resistance [30].

The trajectory of LBMI showed a greater and longer-term increase in children who started GH in the first year of life compared to children who started between age 2–5 years, which is particularly relevant for infants with PWS, as they generally suffer from hypotonia and motor developmental delay. We found that children who started GH in the first year of life had a greater increase in LBMI than children who started between age 2–5 years, which will support the motor development during early childhood.

After 8 years, head circumference SDS was significantly larger in GH-treated children compared to untreated controls. Head circumference has been associated with cognitive functioning [18,31]. GH-treated children scored higher on all the subtests and had a higher Total IQ compared to the untreated controls, but these differences did not reach significance. Children who started GH before the age of 1 year had a significantly higher Total IQ and Vocabulary IQ compared to children who started GH between age 2–5 years, even though the head circumference SDS was similar. These results are in line with our previous study comparing the Total IQ of children who started GH before the age of 2 years with children who started GH at 8 years [19], and in line with a recent study showing a higher Total IQ in children with PWS who started GH before the age of 2 years compared to those who started GH later or did not receive GH at all [32]. Our findings might suggest that starting GH in the first year of life leads to a higher Total IQ score later in childhood, than starting between age 2 and 5 years.

Height SDS and head circumference SDS normalised during 8 years of GH treatment and was significantly higher than in age-matched untreated controls. This normalisation is in line with previous studies [33,34]. Children who started GH treatment before the age of 1 year or between age 2–5 years had a similar height SDS. Two other retrospective studies found that younger age at the start of GH treatment was associated with a higher height SDS [8,9], but we could not confirm this. As 8 years of GH treatment ensures normalisation of height SDS in both age-groups, for height improvement, it is not necessary to start GH in the first year of life.

BMI SDS is not an appropriate parameter to analyse effects of GH in children with PWS, but we added it for comparison with literature data. After 8 years of GH, BMI SDS tended to be lower in children who started GH in the first year compared to those who started between age 2–5 years, which is in line with previous studies [9,35].

Regarding safety parameters, there were no differences in fasting levels of glucose, insulin, triglycerides, diastolic BP SDS, and scoliosis prevalence. Cholesterol was lower in children who started GH in the first year of life. Systolic BP SDS was higher in children who started GH treatment before the age of 1 year, which may be due to the fact that younger children were more anxious. It could be speculated that the higher systolic BP SDS might be an effect of starting GH in early life, but this is less likely, as previous studies in children with PWS or born small for gestational age found that GH treatment had a positive effect on systolic BP [6,36]. As all values were within the normal range, we can conclude that starting GH in the first year of life has no adverse effects on metabolic parameters.

Because all Dutch children with PWS are nowadays treated with GH from a young age, we could only include a small number of age-matched untreated controls. An RCT would have been the first choice to investigate long-term effects of GH in children with PWS in comparison with untreated controls, but it would have been unethical to withhold long-term GH treatment in children with PWS. The data of untreated controls were collected prior to start of GH and these children could, therefore, act as age-matched untreated controls. Besides the early initiation of GH treatment, some other elements of PWS management have changed over time. In our PWS Reference Center, standard Dutch PWS care includes multidisciplinary care with physical therapy and consultations with a dietician for 20 years, but nowadays we emphasise the importance of healthy eating-habits from an early age. Therefore, we assume that there was no bias due to a difference in treatment other than GH, although we cannot exclude that other (unknown) factors associated with the management of PWS might have caused some kind of bias. As PWS is a rare disorder, the number of patients in the subgroups was relatively small. We found differences between the subgroups regarding FM% SDS, FM%, and BMI SDS, but these did not reach significance, probably due to the relatively smaller subgroups. It would have been ideal to match the subgroups for several variables that could have an influence on the outcomes, such as parental education levels, parental BMI, and nutrition habits. However, matching on several variables requires a large number of patients, which is unfeasible in case of a rare disorder. Mentioned variables could potentially have caused some bias, but we consider it as small, as it was an independent medical decision to start GH. One potential variable, parental height, did not differ between the subgroups. In addition, we were not able to randomise the subgroups, as it would nowadays be unethical to withhold GH treatment, even for a few years. A limitation of this study is the difference in pubertal stage between the subgroups after 8 years of GH treatment, although both subgroups had no late-pubertal children. To minimise the effect of the difference in pubertal stage, body composition and metabolic parameters were adjusted for pubertal stage.

In conclusion, starting GH before the age of 5 years in children with PWS leads to better body composition, taller stature, and a larger head circumference after 8 years of treatment compared to age-matched untreated controls with PWS. In addition, we conclude that starting GH in the first year is safe and might be even more optimal than starting between age 2–5 years. Children who started GH in the first year of life had more prominent effects on body composition during the first few years of treatment and had a higher IQ after 8 years. These findings support our hypothesis: The earlier the start of GH treatment, the better the outcomes.

## Figures and Tables

**Table 1:** Baseline characteristics.

	GH-Treated Group vs. GH-Untreated Controls			GH-Treated Subgroups			
	GH-Treated	GH-Untreated	*p*-Value ^b^	A	B	C	*p*-Value ^#^
Number (boys)	84 (44)	22 (11)	0.76	27 (11)	28 (16)	27 (17)	0.102
Genetic subtype N(%)			0.086				0.273
Deletion	44 (54.3)	8 (40.0)		13 (48.1)	14 (51.9)	17 (63.0)	
mUPD	36 (44.4)	10 (50.0)		14 (51.9)	12 (44.4)	10 (37.0)	
ICD	1 (1.2)	2 (10)		0 (0.0)	1 (3.7)	0 (0.0)	
Age at start GH treatment (years)	1.92 (1.20)	-	N.A.	0.75 (0.13)	1.64 (0.37)	3.38 (1.20)	<0.001
Fat mass percentage ^a^	29.7 (0.72)	-	N.A.	29.4 (1.3)	28.2 (1.2)	31.5 (1.2)	0.291
Trunk/peripheral fat ratio ^a^	0.55 (0.02)	-	N.A.	0.52 (0.02)	0.50 (0.02)	0.64 (0.02)	0.001
Height SDS	−2.01 (1.19)	-	N.A.	−1.82 (0.93)	−1.79 (1.17)	−2.42 (1.36)	0.064
Head circumference SDS	−1.05 (0.99)	-	N.A.	−1.42 (0.77)	−1.05 (1.08)	−0.73 (0.99)	0.01
Weight for height SDS	−0.13 (1.57)	-	N.A.	−0.41 (1.06)	−0.57 (1.49)	0.60 (1.85)	0.017
BMI SDS	−0.07 (1.62)	-	N.A.	−0.74 (1.08)	0.39 (1.54)	0.93 (1.72)	<0.001
Target height SDS	0.42 (0.92)	0.16 (0.71)	0.233	0.59 (0.88)	0.43 (0.92)	0.23 (0.97)	0.161

Data are expressed as mean (SD) or N (%). mUPD—maternal uniparental disomy; ICD—imprinting-centre defect; GH—growth hormone; SDS—standard scores; BMI—body mass index; N.A.—Not applicable. ^a^ corrected for sex, expressed as Estimated Marginal Mean (SE); ^b^ *p*-value between the total GH group compared to GH-untreated controls; ^#^ comparison between the lowest (A) and highest (C) tertile of age at start GH.

**Table 2:** Results after 8 years of GH treatment compared to GH-untreated controls.

	GH-Treated Children	GH-Untreated Controls	*p*-Value
	(*n* = 82)	(*n* = 22)	
Age (years)	9.88 (1.19)	10.35 (1.37)	0.117
Puberty (N, %)			0.216
Prepubertal	55 (67.1)	18 (81.8)	
Early pubertal	18 (22.0)	4 (18.2)	
Mid pubertal	9 (11.1)	0	
Late pubertal	0	0	
Fat mass % SDS ^a^	1.90 (0.07)	2.25 (0.15)	0.036
Fat mass percentage ^b^	38.0 (0.89)	46.1 (1.76)	<0.001
Trunk fat vs. peripheral fat ratio ^b^	0.82 (0.01)	0.91 (0.02)	<0.001
Lean body mass SDS ^a^	−1.51 (0.10)	−1.92 (0.20)	0.033
Height SDS	0.29 (1.19)	−2.01 (1.49)	<0.001
Head circumference SDS	0.60 (1.09)	−0.59 (0.75)	<0.001
BMI SDS	1.10 (1.50)	1.63 (1.01)	0.055
Cognitive functioning (SS)			
Block design	4.29 (3.18)	3.19 (2.66)	0.217
Vocabulary	4.94 (2.82)	4.81 (2.07)	0.871
Similarities	6.16 (2.78)	5.06 (2.93)	0.179
Estimated total IQ	70.4 (14.1)	67.3 (10.1)	0.417

Data are expressed as mean (SD). GH—growth hormone; BMI—body mass index; SDS—standard scores based on age and gender; SS—standard scores. ^a^ corrected for height, expressed as Estimated Marginal Mean (SE); ^b^ corrected for height and sex, expressed as Estimated Marginal Mean (SE).

**Table 3:** Results after 8 years of GH treatment in the subgroups.

	A (*n* = 27)	B (*n* = 28)	C (*n* = 27)	*p*-Value ^#^
Age (years)	8.75 (0.19)	9.57 (0.35)	11.33 (0.80)	<0.001
Puberty (N (%))				0.002
Prepubertal	23 (85.2)	20 (71.4)	12 (44.4)	
Early pubertal	4 (14.8)	7 (25.0)	7 (25.9)	
Mid pubertal	0	1 (3.6)	8 (29.6)	
Late pubertal	0	0	0	
Fat mass % SDS	1.81 (0.54)	2.07 (0.65)	2.06 (0.87)	0.212
Fat mass percentage ^a^	36.0 (1.70)	39.5 (1.62)	40.7 (1.78)	0.142
Trunk fat vs. peripheral fat ratio ^a^	0.80 (0.02)	0.82 (0.02)	0.87 (0.02)	0.043
Lean body mass SDS	−1.49 (0.68)	−1.22 (1.19)	−1.33 (1.15)	0.525
Height SDS	−0.08 (0.86)	0.61 (1.25)	0.32 (1.34)	0.199
Head circumference SDS	0.66 (0.89)	0.63 (1.01)	0.53 (1.29)	0.131
BMI SDS	0.80 (1.09)	1.10 (1.04)	1.40 (1.26)	0.064
Cognitive functioning (SS)				
Block design	5.29 (2.93)	4.59 (3.19)	3.58 (3.27)	0.283
Vocabulary	6.71 (2.56)	5.50 (2.72)	3.63 (2.54)	0.012
Similarities	7.29 (2.36)	6.57 (2.31)	5.26 (3.26)	0.148
Estimated total IQ	78.1 (12.6)	72.8 (12.9)	64.8 (14.5)	0.043

Data are expressed as mean (SD). GH—growth hormone; BMI—body mass index; SDS—standard scores based on age and gender; SS—standard scores. ^a^ corrected for sex and pubertal stage, expressed as Estimated Marginal Mean (SE); ^#^ comparison between the lowest (A) and highest (C) tertile of age at start GH.

**Table 4:** Safety parameters after 8 years of GH treatment between the subgroups.

	A (*n* = 27)	B (*n* = 28)	*C* (*n* = 27)	*p*-Value ^#^
IGF-1 SDS	1.92 (0.57)	2.13 (0.68)	2.06 (0.94)	0.516
Average IGF-1 SDS during 8 years of GH treatment	2.36 (0.58)	2.59 (0.45)	2.17 (0.89)	0.349
Fasting glucose ^a^ (mmol/L)	4.94 (0.08)	4.84 (0.08)	4.84 (0.09)	0.469
Fasting insulin ^a^ (pmol/L)	73.4 (10.1)	73.1 (10.1)	76.0 (10.3)	0.979
HOMA-IR ^a^	2.32 (0.34)	2.33 (0.34)	2.44 (0.35)	0.961
Triglycerides ^a^ (mmol/L)	0.84 (0.07)	0.91 (0.07)	0.87 (0.07)	0.440
Total cholesterol ^a^ (mmol/L)	4.13 (0.15)	4.32 (0.15)	4.55 (0.16)	0.041
HDL cholesterol ^a^ (mmol/L)	1.58 (0.07)	1.56 (0.07)	1.60 (0.07)	0.944
LDL cholesterol ^a^ (mmol/L)	2.49 (0.15)	2.55 (0.15)	2.74 (0.16)	0.207
Systolic blood pressure SDS	0.72 (0.89)	0.32 (1.05)	−0.06 (0.88)	0.002
Diastolic blood pressure SDS	0.20 (0.51)	0.33 (0.67)	0.35 (0.80)	0.395
BA/CA ratio	1.04 (0.10)	1.08 (0.14)	1.05 (0.11)	0.731
Scoliosis	14 (51.9)	20 (71.4)	18 (66.7)	0.668

Data are expressed as mean (SD) or N (%). HOMA-IR—Homeostatic Model Assessment for Insulin Resistance; HDL—high-density lipoprotein; LDL—low-density lipoprotein; SDS—standard scores BA/CA ratio—bone-age-to-calendar-age ratio ^a^ corrected for pubertal stage, expressed as Estimated Marginal Mean (SE); ^#^ comparison between the lowest (A) and highest (C) tertile of age at start GH.

## Data Availability

The datasets generated during and/or analysed during the current study are not publicly available but are available from the corresponding author on reasonable request.
